# Life-threatening amiodarone-induced thyrotoxicosis – Personalized approach to radical treatment

**DOI:** 10.1016/j.heliyon.2024.e34850

**Published:** 2024-07-18

**Authors:** Marta Opalińska, Jacek Pantofliński, Grzegorz Sokołowski, Dorota Pach, Marta Kostecka-Matyja, Katarzyna Żabicka, Bartosz Partyński, Małgorzata Kieć-Klimczak, Anna Sowa-Staszczak, Monika Buziak-Bereza, Aleksandra Gilis-Januszewska, Alicja Hubalewska-Dydejczyk

**Affiliations:** a Chair and Department of Endocrinology, Jagiellonian University Medical College, ul. Jakubowskiego 2, 30-688, Krakow, Poland; b Nuclear Medicine Unit, Endocrinology, Oncological Endocrinology and Nuclear Medicine Department, University Hospital, ul. Jakubowskiego 2, 30-688, Krakow, Poland

**Keywords:** Thyrotoxicosis, AIT, Amiodarone, Salvage therapy, 131-I

## Abstract

**Objective:**

Amiodarone is an iodine-rich molecule and an effective antiarrhythmic drug. It is a first-line treatment for patients with life-threatening ventricular arrhythmias and for prevention in patients at high risk. The use of amiodarone may cause serious adverse effects such as pharmacotherapy-resistant, life-threatening amiodarone-induced thyrotoxicosis (AIT)leading to rapid deterioration of the patient's condition.

According to the European Thyroid Association (ETA) guidelines, emergency thyroidectomy is the first-line treatment option in these cases. ; however, is not always feasible in the clinical setting due to the high anesthetic risk.

We aimed to assess the clinical course and results of urgent thyroidectomy and 131-I therapy in patients with severe AIT with worsening of cardiac status.

**Methods:**

Retrospective analysis of the clinical course and outcomes of life-threatening AIT refractory to pharmacotherapy in patients hospitalized at a tertiary endocrinology center between 2014 and 2022.

**Results:**

An electronic database search identified 75 patients hospitalized for severe AIT. At the time of AIT diagnosis, median Thyroid-stimulating hormone (TSH) concentration was 0.001 mIU/L (range 0.001–0.35), fT4 63.2 pmol/L (range 9.0 - >100), and fT3 10.2 pmol/L (range 3.8–49.3). All patients received optimal conservative treatment. Among them, 20 required urgent radical therapy due to worsening arrhythmias and/or AIT-related heart failure. In this group, 6 patients died before any radical treatment was applied, 6 underwent total thyroidectomy, while 8 patients were successfully treated with 131-I (in 6 cases after rhTSH stimulation). The median dose of 131-I used for the therapy was 784MBq (range 627–860). The decision to treat with 131-I despite low but detectable 131-I uptake (median value 6 %) was made in cases of significant contraindications to anesthesia due to refractory ventricular arrhythmias, exacerbation of severe heart failure unresponsive to cardiac treatment, myocardial infarction during AIT course, massive pulmonary embolism.

**Conclusion:**

The decision regarding the optimal time and type of radical treatment of AIT refractory to pharmacotherapy is critical for patients management and should not be delayed. Urgent therapy with 131-I may be an effective therapeutic option in patients who are unsuitable for thyroidectomy due to the high risk of anesthesia.

## Highlights

• AIT refractory to the best medical treatment becomes a challenge for clinicians.
• The time and type of AIT refractory to treatment is crucial and can be life-saving.
• The first-line option (thyroidectomy) is not always feasible due to the high cardiac risk.
• Urgent 131-I therapy of severe AIT shows high efficacy and good safety profile.

## 1 Introduction

Amiodarone is a widely used therapeutic option for the management of life-threatening ventricular arrhythmias and for the prevention of ventricular tachyarrhythmias in high-risk patients. It is also widely used to treat supraventricular tachyarrhythmias such as atrial fibrillation [1].

The early effect of amiodarone on serum thyroid hormone concentrations is well known. During the first month of amiodarone use, a 20–40 % increase in free thyroxine (fT4) and a 30 % decrease in free triiodothyronine (fT3) are often observed, accompanied by a slight increase in TSH- [1]. Normalization of TSH concentration is usually seen up to 6 months, while fT4 values remain near the upper limit of normal or slightly elevated and fT3 decreases to the lower limit of normal or even below this range [2].

In 15–20 % of patients, the use of amiodarone leads to thyroid dysfunction requiring medical intervention [3]. Amiodarone-induced hypothyroidism is quite common, especially in regions deficient in iodine; however, it is usually easily managed with replacement therapy. Amiodarone-induced thyrotoxicosis (AIT) is less common, constitutes 3–9% [3,4] of thyroid dysfunction cases, and may lead to life-threatening complications. Furthermore, management of AIT is often considered a clinical challenge resulting from the prolonged biological half-life of amiodarone (2 weeks–3 months) and the large amount of iodine in amiodarone molecules (200 mg of amiodarone releases approximately 6 mg of iodine) [5]. Because of this, post-amiodarone side effects may occur or persist for a long period of time after discontinuation of treatment [5].

There are 2 main pathomechanisms of AIT. Type 1 AIT usually occurs in patients with pre-existing thyroid disease. Type 2 AIT is associated with destructive thyroiditis that leads to excessive release of thyroid hormones and occurs in patients with initially normal thyroid function [6]. According to epidemiological studies, type 1 AIT is more common in regions deficient in iodine, while type 2 AIT is most common in iodine-replete environments [7]. The differential diagnosis of both types of AIT can be difficult, and in such cases, a clinical diagnosis of mixed/undefined form of AIT is accepted [8].

Complications of prolonged and refractory to treatment AIT, result in increased mortality, especially in elderly patients with heart failure and life-threatening arrythmias. It should be noted that this high-grade thyrotoxicosis also affects the cardiovascular system by exacerbating heart failure and arrhythmias [9]. A multicenter study that analyzed the causes of thyroid storm in French patients showed that AIT was the most common cause among patients hospitalized in 31 intensive care units, with a mortality rate of 17 % in intensive care units and 22 % at 6 months [10].

The main goal of AIT treatment is to achieve euthyroidism as soon as possible. Short-term therapeutic options for AIT include amiodarone cessation, thioamide administration, sodium perchlorate, glucocorticoids, and plasmapheresis [11]; however, in some cases, this treatment is ineffective.

In AIT which is refractory to medical treatment, persistent thyrotoxicosis with progressive deterioration of cardiovascular function requires urgent resolution.

Another key clinical issue is the possibility of amiodarone withdrawal. The decision to discontinue amiodarone should take into account the possible clinical benefit of amiodarone use in life-threatening arrhythmias, the risk of long-term exposure to excess thyroid hormones, and the type of AIT. The withdrawal of amiodarone unblocks T3 receptors in myocardial cells and may exacerbate life-threatening arrhythmias. Drug withdrawal can be especially useful in type 1 AIT and in mixed/indeterminate types of AIT [11].

There are two main treatment options for the management of radical thyrotoxicosis: total thyroidectomy or 131-I therapy. In some cases of AIT, the first option may not be possible due to the high risk associated with anesthesia, while the second option is often hampered by low 131-I uptake.

We aimed to assess the clinical course and results of urgent thyroidectomy or 131-I therapy in patients with severe AIT who were hospitalized due to worsening of circulatory insufficiency and/or arrythmias.

## 2 Materials and methods

**Fig. 1:**
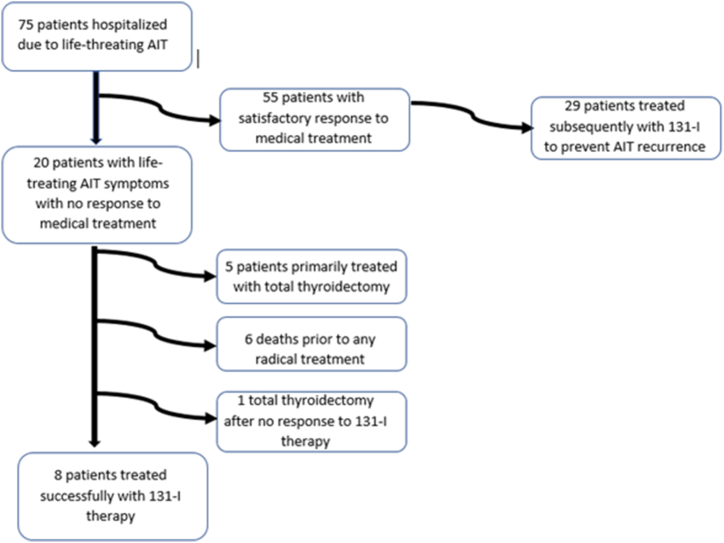
Consort diagram for the final study population.

An electronic database search was conducted among patients hospitalized due to AIT at a tertiary endocrinology center between 2014 and 2022. Of these, patients who required urgent treatment consisting of thyroidectomy or 131-I therapy (with or without recombinant human thyrotropin (rhTSH) stimulation) due to aggravation of arrhythmias and/or AIT-related heart failure were selected. Finally, demographic data, clinical presentation of AIT, diagnostic tests, choice of treatment method, response to routine treatment, and results of urgent therapy were evaluated. The consort diagram for the study population is presented in Fig. 1.

### 2.1 AIT identification and outcome definition

Amiodarone-induced thyrotoxicosis was diagnosed if thyroid dysfunction was related to the use of amiodarone up to 3 months before the onset of thyrotoxicosis. Possible misdiagnosis due to “euthyroid sick syndrome” or the effects of medication (i.e., heparin, dopamine, glucocorticoids) [12] were excluded based on a patient’s medical history. The results of diagnostic procedures such as thyroid ultrasound (USG) (with assessment of thyroid echogenicity, presence of thyroid nodules, the vascularity in ultrasound color Doppler, and estimation of thyroid volume), thyroid scintigraphy, and presence of thyroid specific antibodies were recorded. The reference values for all laboratory tests are shown in Table 1.

**Table 1:** Results of thyroid function assessment and concentration of anti-thyroid antibodies at the time of AIT diagnosis, liver enzymes, and NT-proBNP serum concentrations during hospitalization in the whole group of patients.

Variable	Median (range)	Normal range
TSH at diagnosis [mIU/L]	0.001 (0.001–0.35)	0.27–4.20
Free T4 at diagnosis [pmol/L]	63.17 (9.0 – >100)	12.0–22.0
Free T3 at diagnosis [pmol/L]	10.5 (3.8–49.30)	3.1–6.8
TRAb, [U/L]	0.6 (0.10–11.80)	0.0–1.0, pathology >2.0
aTPO, [IU/mL]	11,58 (0.00–199.00)	0.0–34.0
aTg, [IU/mL]	14.1(0.00–441.0)	0.0–115.0
NT-proBNP [pg/mL]	2289.00 (218–25,000)	0.0–125.0
AST [U/L]	22.0 (11.0–299.0)	10–35
ALT [U/L]	34.0 (9.0–489.0)	10–35

TSH - Thyroid-stimulating hormone, T4 – thyroxine, T3 – triiodothyronine, TRAb - TSH receptor antibodies, aTPO - anti-thyroid peroxidase antibodies aTg - thyroglobulin antibodies NT-proBNP - N-terminal prohormone of brain natriuretic peptide, AST - Aspartate transaminase, ALT - alanine aminotransferase.

AIT was defined as suppression of TSH with elevated or normal T4 and/or T3 levels after exclusion of sick euthyroid syndrome and drug effects. Type 1 AIT was defined by the presence of nodular thyroid goiter or diffuse goiter >20 g or a positive TRAb (TSH receptor antibodies) concentration. AIT type 2 was defined as the presence of a normal thyroid gland or a small diffuse goiter <20 g with a negative concentration of TRAb. In cases not qualifying as type 1 or 2, a mixed/undefined form of AIT was diagnosed.

The first day of AIT was assumed to be the date on which the constellation of results typical for AIT was identified for the first time. In the case of surgical treatment, we considered the day of surgery to be the day of recovery. In the case of patients treated with 131-I or with pharmacotherapy alone, the day of recovery was considered the day of fT3 and fT4 normalization. Thyroid scintigraphy was performed with a 131-I dose of 6MBq. Radioiodine uptake (RAIU) was measured 24 h after 131-I application (24 h RAIU). A low 24 h RAIU was considered as below 4 % in the context of AIT.

In the case of rhTSH stimulation before 131-I therapy, patients received 0.9 mg of thyrotropin alfa intramuscularly 24 h before 131-I therapy.

The cardiac status was evaluated using left ventricular ejection fraction (LVEF, measured by echocardiography), NT-proBNP concentration, and appearance of hemodynamic instability. Events related to the duration of hospitalization and the appearance of clinical complications were recorded.

The successful outcome of AIT treatment was considered as achievement of permanent euthyroidism or hypothyroidism.

### 2.2 Statistical analysis

Demographic and clinical characteristics were analyzed by producing frequency tables for categorical variables and by calculation of the median and range for continuous variables due to the small cohort size and non-normal data distribution. Categorical variables were provided as numbers (n) and percentages (%). Statistical analyses comparing groups of patients were performed using non-parametric tests, including the Wilcoxon rank-sum test for continuous variables and Fisher’s exact test for binary variables. A p-value <0.05 was considered statistically significant.

IBM SPSS Statistics for Windows, version 27 (IBM Corp., Armonk, NY, USA) was used for the statistical analysis.

## 3 Results

In total, 75 patients who met the inclusion criteria were enrolled in the study.

### 3.1 Incidence and baseline characteristics at the time of AIT diagnosis

There were significantly more males than females among the patients eligible for the study: 56 males (74.7 %) vs 19 females. The median age at the time of diagnosis was 64 years (range 31–93). Significant cardiovascular risk in patients included: a history of sudden cardiac arrest in 11 (14.7 %) patients, coronary artery disease in 38 (50.7 %) cases, and arrhythmias, mainly supraventricular in 70 (93.3 %) patients. A cardioverter defibrillator or cardiac pacemaker was present in 23 (30.7 %) and 5 (6.7 %) patients, respectively. Detailed baseline characteristics and clinical data of the patients are presented in Table 2.

**Table 2:** Patient Characteristics and Clinical Presentation at diagnosis.

Patient Characteristics and Clinical Presentation	
Variable	Median (range)
Age, years	64,0 (31.0–93.0)
BMI, kg/m2	27.2 (19.5–43.6)
Ejection fraction, %	30.0 (10.0–67.0)
Duration of amiodarone therapy before AIT, months	21.3 (0.1–48.0)
Variable	No of patients (%)
Sex, Male	56 (74,7 %)
Hyperthyroidism before amiodarone use (unnoticed at time of amiodarone implementation)	5 (12.8 %)
Diabetes mellitus	37 (49.3)
Chronic kidney disease	16 (21.3)
Pulmonary disease	11 (14.7 %)
Arrhythmias	70 (93.3)
Coronary artery disease	38 (50.7)
Myocardial infarction	20 (26.7)
Cardiac pacemaker	5 (6.7)
Cardioverter-defibrillator	23 (30.7)
Smoking	36 (49.3)
Family history of thyroid disease	9 (12.0)
Main clinical symptoms during hospitalization	No of patients (%)
Fatigue	35 (46.7)
Dyspnea	19 (25.3)
Tachycardia	11 (15.1)
Hypertension despite treatment	4 (5.3)
Arrythmias	35 (46.6)
Decompensated heart failure	15 (20.0)
Classical thyrotoxicosis symptoms (weight loss, tremor, palpitations)	39 (52.0)
Gastrointestinal symptoms	16 (21.3)
Acute coronary syndromes and thoracic pain	20 (26.7)

At the time of the diagnosis of AIT, the median TSH was 0.001 mIU/L (range 0.001–0.35), fT4 63.2 pmol/L (range 9.0 – >100), and fT3 10.2 pmol/L (range 3.8–49.3). The median concentrations of antithyroid antibodies were: TRAb 0.6 U/L (range 0.1–11.8), aTPO 11.6 IU/mL (0.00–199.00), aTg 14.1 IU/mL (range 0.0–441.0). The median NT-proBNP was 2289.0 pg/mL (range 218–25,000). A summary of the laboratory results is provided in Table 1.

Most of the patients were symptomatic and had classic symptoms of thyrotoxicosis, such as palpitations, weight loss, sweating, and heat intolerance. The most prominent cardiac and endocrine symptoms related to AIT observed during hospitalization are provided in Table 2.

The median thyroid volume was 23.5 mL (range 8.8–152.36). In 46 cases, hypoechogenicity typical of thyroiditis and/or decreased tissue vascularity was observed on the power Doppler. Nodular goiter was found in 33 cases. Both pathologies overlapped in 11 patients. In 7 cases, the blood flow was not assessed by USG. Five (12.8 %) patients had been diagnosed with thyroid dysfunction prior to the use of amiodarone, but they received amiodarone in the emergency unit without prior consideration of thyroid history. During thyroid evaluation, pathological values of TSH receptor antibodies (TRAb) were observed in 7 patients.

### 3.2 Amiodarone use

One patient without previous thyroid dysfunction received a single dose of amiodarone. The rest of the patients received prolonged amiodarone treatment for a median time of 21.3 months (range 0.1–48.0). Amiodarone was discontinued in 73 (97.0 %) patients after the diagnosis of AIT (following consultation with a cardiologist), except one patient with a 3-year history of sudden cardiac arrest due to ventricular tachycardia, recurrent episodes of VT despite the use of several antiarrhythmic drugs (b-blockers, verapamil, xylocaine, mexiletine, propafenone), and repeated RF ablations. In this case, it was decided to maintain amiodarone with concomitant thyreostatic treatment.

### 3.3 Medical management of thyrotoxicosis

Hyperthyroidism during hospitalization was treated with antithyroid drugs (ATD) in all 75 (100 %) cases, mainly by intravenous injections (in 71 cases). Treatment with thiamazole was initiated in all cases. In 6 patients, thiamazole was discontinued due to adverse effects (neutropenia, skin rash, deterioration of liver function), and the administration of propylthiouracil was initiated. Glucocorticosteroids were used in 60 (80 %) patients, sodium perchlorate in 38 (50.7 %), lithium carbonate in 10 (13.3 %) and albumin in 16 (21.3 %) cases. The sequence of medication use depended on the individual course of the disease, including the type of AIT. Usually, ATD was initiated upon suspicion of type 1 AIT, while ATD, glucocorticosteroids, and/or sodium perchlorate were initiated upon suspicion of type 2 AIT as well as in the case of mixed/undefined type. If there was no clinical or biochemical effect, additional medication was added to the therapy. The drug regimens used during hospitalization are shown in Table 3. The average and maximumdoses of the medication used in AIT treatment are provided in Table 4.

**Table 3:** AIT medical management during hospitalization.

Sets of medicines used	No of patients (%)
ATD	13 (17.3)
ATD + steroids	20 (26.7)
ATD + steroids + sodium perchlorate	20 (26.7)
ATD + steroids + sodium perchlorate + lithium carbonate	5 (6.7)
ATD + steroids + sodium perchlorate + albumin	9 (12.0)
ATD + steroids + albumins	3 (4.0)
ATD + steroids + lithium carbonate	1 (1.3)
ATD + steroids + sodium perchlorate + lithium carbonate + albumins	4 (5.3)
Discontinuation of amiodarone after diagnosis of AIT	73 (97)

ATD – antithyroid drugs.

**Table 4:** Average and maximal doses of drugs used for AIT treatment.

	Average daily dose	Maximal daily dose
ATD: - Thioamide - Propylthiouracil	60 mg p.o/80 mg i.v 300 mg	160 mg i.v.* 300 mg
Steroids: - oral methylprednisolone - intravenous methylprednisolone - prednisolone	16 mg 125 mg 40 mg	32 mg 250 mg 80 mg
Sodium perchloride	30’/600 mg	60’/1200 mg
Lithium carbonate	750 mg	750 mg
Albumins	400 mg	600 mg

### 3.4 Therapy outcome

The median duration of hospitalization time for all AIT patients was 17 days (range 2–107). During this time, urgent radical treatment was indicated in 20 (26.7 %) patients. These indications included severe thyrotoxicosis refractory to medical treatment with worsening cardiac condition, intolerance or contraindication to ATD, and in a single case, severe metabolic side-effects of prolonged glucocorticoid use (Table 5). The comparison of thyroid function assessment at the time of AIT diagnosis as well as concentration of anti-thyroid antibodies, liver enzymes, and NT-proBNP serum concentrations in patients who required urgent radical therapy are shown in Table 6.

**Table 5:** Clinical indications for urgent AIT radical treatment.

	131-I, no of cases	Thyroidectomy, no of cases	Death, no of cases
ATD intolerance (drug-induced liver failure, neutropenia, skin rush) accompanying severe thyrotoxicosis with cardiac status deterioration	4		
Contraindication to anesthesia due to poor cardiological status (SVT exacerbating severe cardiac insufficiency non-responding to cardiology treatment, MI during AIT treatment, massive pulmonary embolism)	4		
SVT non-responding to cardiology treatment		2	
Exacerbation of cardiac insufficiency during AIT treatment		2	
Increasing side effects of steroid therapy		1	
Progressive pulmonary hypertension		1	
Multiple organ failure			4^a^
Sudden cardiac death			2

a Including one case of amiodarone-induced severe pulmonary fibrosis.

**Table 6:** Comparison of thyroid function assessment and anti-thyroid antibodies concentration, liver enzymes, and NT-proBNP serum concentrations in patients who underwent urgent radical therapy.

Variable	Patients treated with thyroidectomy (no 6)	Patients treated with 131-I (no 8)	Patients who died before any radical treatment (no 6)	p
TSH at diagnosis [mIU/L]	0.001 (0.001–0.22)	0.001 (0.001–0.19)	0.001 (0.00–0.35)	0.643
Free T4 at diagnosis [pmol/L]	100 (25,5 - >100)	100 (25 - >100)	100 (63 - >100)	n/a
Free T3 at diagnosis [pmol/L]	24.9 (4.8–35.8)	14,66 (5.19–35.8)	14.23 (6.36–28.19)	0.517
TRAb, [U/L]	0.35 (0.3–0.6)	0.7 (0.4–2.1)	0.5 (0.3–1.3)	0.671
aTPO, [IU/mL]	9.72 (0.00–26.82)	14.43 (8.42–26.82)	15.48 (0.00–27.25)	0.325
aTg, [IU/mL]	16.4 (0.00–59.8)	12.75 (0.00–43.8)	13.2 (0.00–22.7)	0.435
NT-proBNP [pg/mL]	524 (192–2729)	2370 (455.4–25,000)	13,011 (1386–25000)	0.182
ALT [U/L]	49 (29–97)	28 (15–76)	104 (15–489)	0.341
AST [U/L]	20 (18–39)	19 (14–54)	56 (16–299)	0.842

TSH - Thyroid-stimulating hormone, T4 – thyroxine, T3 – triiodothyronine, TRAb - TSH receptor antibodies, aTPO - anti-thyroid peroxidase antibodies aTg - thyroglobulin antibodies NT-proBNP - N-terminal prohormone of brain natriuretic peptide, AST - Aspartate transaminase, ALT - alanine aminotransferase.

Among the 20 patients whose condition deteriorated, 6 patients died before any radical treatment (thyroidectomy or 131-I therapy) was started, despite having received the best possible medical treatment, including plasmapheresis in 1 case. Total thyroidectomy was performed successfully in 6 patients; 1 patient developed cardiogenic shock in the perioperative period. Nine patients who were disqualified from surgery due to severe heart failure, life-threatening ventricular arrhythmias, and massive pulmonary embolism were treated with urgent 131-I therapy. Six of them (6/9) received 0.9 mg of rhTSH intramuscularly 24 h before the therapy due to low RAIU. The median dose of 131-I used for the first therapy was 784MBq (range 627–860). In 4 patients, a single dose of 131-I was successful in achieving hypothyroidism. Repeat therapy with 131-I was required in 4 patients due to a recurrence of thyrotoxicosis (with less severe symptoms). Among this group, 3 patients required a third dose of 131-I treatment to achieve hypothyroidism. In all cases of repeated 131-I therapy, the doses were 784MBq and were administered without the use of rhTSH. In one of these patients, there was no improvement after 131-I therapy and the patient finally underwent a thyroidectomy.

In the remaining cases (55/75), thyrotoxicosis was successfully controlled with the use of conservative management. Among these patients, 29 were subsequently treated with 131-I to radically cure hyperthyroidism or prevent recurrence of AIT in the future. The relevant clinical data for these patients are provided in the Supplementary Materials. In 48 out of 55 (87.3 %) patients who did not require urgent AIT therapy, a treatment regimen using ATD in alternation with glucocorticoids and/or sodium perchlorate was sufficient.

**Fig. 2:**
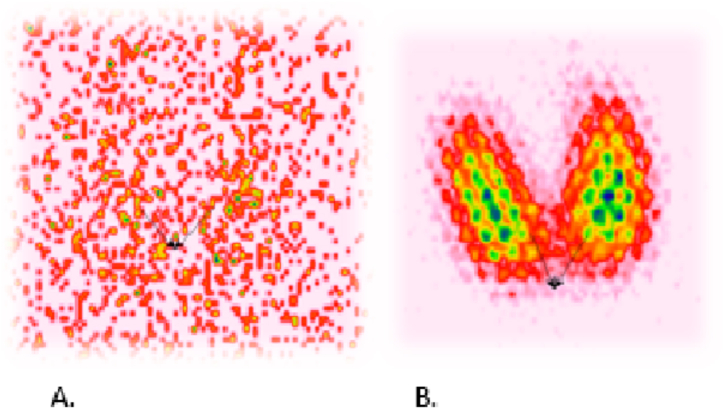
Low RAIU in pre-treatment (A) and good RAIU in post-treatment scintigraphy (B) (after rhTSH i.m. injection 24 h before therapy) in the patient with life-threatening AIT complicated by massive pulmonary embolism, successfully treated with 131-I.

For all patients treated with 131-I, the median time from the last use of amiodarone to the 131-I therapy was 7.00 months (range 1–27). The median 24 h RAIU was 6.0 %, measured after administration of 6 MBq of 131-I. The example of RAIU in pre- and post-treatment scintigraphy in the patient with a good response to 131-I after rhTSH stimulation is shown in Fig. 2. The median time to achieve euthyroidism or hypothyroidism was 112 days (range 30–270).

## 4 Discussion

Refractory AIT overlapping with severe heart failure, pre-existing arrhythmias, or unstable coronary artery disease can lead to a life-threatening condition. In our study, 20 of 75 (26.7 %) patients hospitalized for exacerbation of cardiac symptoms in the course of AIT required urgent therapy, despite having received the best available medical treatment (ATD, glucocorticosteroids, sodium perchlorate, lithium carbonate, albumin, plasmapheresis). Among this group, 6 patients underwent successful thyroidectomy and 8 patients were successfully treated with urgent 131-I therapy. Six patients died before any radical treatment was applied.

According to the guidelines of the European Thyroid Association (ETA), emergency total thyroidectomy is recommended in cases of cardiac deterioration due to thyrotoxicosis which do not respond to medical treatment, the appearance of adverse effects of AIT medical therapy, and in patients who may need amiodarone treatment in the future [11]. This procedure may not always be possible due to the risks associated with anesthesia. In such cases, radical therapy using 131-I treatment is possible, optionally with (off-label) rhTSH stimulation in the case of low RAIU. Although thyroidectomy should be considered first-line treatment according to ETA guidelines, this procedure has well-documented perioperative risks [12]. For this reason, our objective was to summarize our experience with urgent 131-I therapy. Although it may be associated with a delayed clinical response compared to thyroidectomy, 131-I therapy has a better risk-benefit profile and should be considered in certain patients with high surgical risk.

Our cohort consisted of predominantly middle-aged and elderly patients with clinically significant cardiac dysfunction and was therefore, a high-risk population at baseline. Among patients who underwent thyroidectomy, rapid resolution of thyrotoxicosis was documented, although a transient worsening of the cardiac condition and a single case of successfully treated cardiac arrest were observed. It is worth mentioning, that the data available in the literature tend to show a long-term improvement in LVEF after thyroidectomy [13], however, thyroidectomy complications in AIT patients were reported in 41 % of cases [12], and this prevalence is greater than in patients with Graves’ disease [14] or toxic multinodular goiter [15]. Among the most serious complications, stroke or respiratory failure were with a frequency of 5.9 % in both cases. The overall mortality in these patients was 5.9 % [12]. In our cohort, no perioperative deaths occurred due to careful qualification for surgery and consideration of 131-I therapy in patients with a high risk associated with anesthesia. However, it should be noted that 6 patients died before the decision was taken regarding the optimal time and type of radical treatment to be used.

In patients with high perioperative risk, effective treatments with fewer adverse effects are still being investigated. As reported in the literature, plasmapheresis [16,17], plasma exchange [18,19], thyroid arterial embolization techniques [20], or atrioventricular node ablation [21] may be successful therapeutic options in the management of AIT. Among these, plasmapheresis and plasma exchange are the most documented, and both can be an option prior to surgery for the rapid reduction of free thyroid hormones and excess iodine. Unfortunately, their effects are rather transient and their use may be associated with an escalation of circulatory instability [22]. The only patient in our cohort who qualified for plasmapheresis prior to urgent thyroidectomy died before surgery due to exacerbation of heart failure. Endovascular embolization techniques appear to be relatively effective and safe [20], but their availability is limited.

Amiodarone-induced thyrotoxicosis remains a diagnostic and therapeutic challenge, and we tried to distinguish AIT subtypes in all cases. Even with a primary suspicion of type 1 AIT, not all patients respond well to ATD, and often require combined therapy with ATD and glucocorticoids or sodium perchloride [23]. However, standard therapy consisting of ATD, glucocorticoids and/or sodium perchloride was effective in 88.9 % of patients, therfore, the ineffectiveness of such a drug mixture may indicate a higher risk of deterioration and the need for subsequent radical treatment.

The decision to continue or discontinue amiodarone after the diagnosis of AIT is often difficult and there is no clear consensus in this regard [11]. Amiodarone and its metabolites have a long half-life, so there is no immediate benefit of its withdrawal. Furthermore, the continuation of amiodarone can be beneficial in the case of life-threatening arrhythmias, so its sudden withdrawal may cause an initial exacerbation of symptoms of thyrotoxicosis [23]. In our cohort, we discontinued amiodarone in the vast majority of patients (97 %) and no sudden worsening of clinical symptoms was observed.

According to previously published data, recurrence of AIT after amiodarone reintroduction is 4 times more frequent in patients with type 1 AIT when compared to patients with type 2 AIT [24]. For this reason, thyroid ablation with 131-I should always be considered before reintroducing amiodarone in patients with a history of type 1 AIT. In one of the previous studies, even patients with a 24 h RAIU of 1.3 % ± 0.9 responded well to 131-I therapy, although the response time to 131-I therapy was significantly longer than in the group with 24 h RAIU equal to 14.1 % ± 0.3 [25]. In the case of an urgent need for 131-I therapy, the situation is more complicated. 131-I therapy is considered ineffective and unfeasible in patients with AIT due to the frequently observed low RAIU. Furthermore, a possible delay in response to treatment (when compared to surgery) may pose an additional high risk. Our results indicate that 131-I therapy may be an effective option, despite low RAIU, and appears to be safe. One of the well-known methods for increasing RAIU is the use of rhTSH stimulation [26]. In theory, the use of rhTSH may be associated with a sustained increase in serum thyroid hormone concentrations, which may exacerbate cardiac failure. In our study, no significant cardiac symptoms were observed among the patients who qualified for urgent 131-I therapy and who received a single dose of rhTSH 24 h before 131-I treatment, which confirmed the safety of this procedure. We would like to stress that rhTSH was used only in patients with low RAIU as seen on pre-treatment scintigraphy. The results of the 131-I therapy obtained in our cohort were satisfactory; only a single patient did not respond to the 131-I therapy and was finally referred for thyroidectomy. Due to the partial response to 131-I treatment in patients who had a recurrence of thyrotoxicosis during the follow-up period, it was possible to repeat 131-I therapy during the period of cardiac stability.

The mechanisms leading to resistance to AIT treatment are largely known, but it is not entirely clear what factors may determine the differential response to treatment. Among other things, it is unclear whether amiodarone itself or its main metabolite desethylamiodarone (formed by metabolism of the drug by cytochrome P4503A) more strongly affects thyroid function. An increased ratio of desethylamiodarone to amiodarone was observed in patients with AIT (prior to the development of thyroid dysfunction), so perhaps it could be a potential predictive marker for the development of AIT [27].

The main limitations of our study are its retrospective design and the small sample of patients undergoing urgent radical treatment exposes our findings to bias, so a multicenter clinical trial could enhance the value of 131-I therapy in this challenging clinical setting. Due to the small sample size and the difficulty with accurate assessment of the AIT type (performed on the basis of available medical records), we were unable to compare treatments in the different subtypes of AIT. Furthermore, the retrospective design of the study limited our ability to accurately compare forms of radical therapy in terms of effectiveness and the associated risks. However, the effectiveness and the good safety profile of 131-I salvage therapy are encouraging. The decision regarding the optimal time and form of radical AIT therapy should take into account the risks and benefits of treatment, which requires an individual approach depending on the severity of thyrotoxicosis, cardiac status, and dysfunction of other organs. In clinical settings, the therapeutic plan should include the coordinated efforts of endocrinologists, thyroid surgeons, cardiologists, and anesthesiologists.In summary, the need for urgent AIT therapy due to a life-threatening cardiac condition affects a relatively small number of patients. The majority of patients, whose condition worsened, even after receiving the best medical treatment required a prompt decision on radical treatment, as its delay could be associated with an unfavorable outcome. The decision to undergo surgery or 131-I therapy should depend on the patient's clinical condition.

In our study, thyroidectomy resulted in rapid resolution of thyrotoxicosis in all analyzed patients. A similar final outcome was observed among patients treated with urgent 131-I therapy. Low RAIU values observed frequently in the course of AIT can be overcome using rhTSH, which in our patients was considered safe. The prevalence of complications in patients after 131-I therapy was negligible.

## Ethics approval

The research was carried out ethically according to the Declaration of Helsinki of the World Medical Association. The study protocol was approved by the Local Ethics Committee of the Jagiellonian University in Krakow (approval no 1072.6120.181.2022).

## Statement of informed consent

In accordance with national legislation and institutional requirements, written informed consent for participation in this study was not required.

## Funding

None.

## Data availability statement

All data generated or analyzed during this study are included in this article [and/or] its supplementary material files. Further inquiries can be directed to the corresponding author.

## CRediT authorship contribution statement

**Marta Opalińska:** Writing – review & editing, Writing – original draft, Software, Methodology, Investigation, Formal analysis, Data curation, Conceptualization. **Jacek Pantofliński:** Resources, Data curation. **Grzegorz Sokołowski:** Writing – review & editing, Resources, Data curation. **Dorota Pach:** Resources, Data curation. **Marta Kostecka-Matyja:** Resources, Data curation. **Katarzyna Żabicka:** Resources, Project administration, Data curation. **Bartosz Partyński:** Resources, Project administration, Data curation. **Małgorzata Kieć-Klimczak:** Resources, Data curation. **Anna Sowa-Staszczak:** Resources, Data curation. **Monika Buziak-Bereza:** Resources, Data curation. **Aleksandra Gilis-Januszewska:** Resources, Data curation. **Alicja Hubalewska-Dydejczyk:** Writing – review & editing, Writing – original draft, Supervision, Resources, Project administration, Methodology, Investigation, Data curation, Conceptualization.

## Declaration of competing interest

The authors declare that they have no known competing financial interests or personal relationships that could have appeared to influence the work reported in this paper.

## References

[1] Hamilton D Sr, Nandkeolyar S., Lan H., Desai P., Evans J., Hauschild C., Choksi D., Abudayyeh I., Contractor T., Hilliard A. (2020 Dec). Amiodarone: a comprehensive guide for clinicians. Am. J. Cardiovasc. Drugs.

[2] Basaria S., Cooper D.S. (2005 Jul). Amiodarone and the thyroid. Am. J. Med..

[3] Farhan H., Albulushi A., Taqi A., Al-Hashim A., Al-Saidi K., Al-Rasadi K., Al-Mazroui A., Al-Zakwani I. (2013 Nov 29). Incidence and pattern of thyroid dysfunction in patients on chronic amiodarone therapy: experience at a tertiary care centre in oman. Open Cardiovasc. Med. J..

[4] Martino E., Safran M., Aghini-Lombardi F., Rajatanavin R., Lenziardi M., Fay M., Pacchiarotti A., Aronin N., Macchia E., Haffajee C., Odoguardi L., Love J., Bigalli A., Baschieri L., Pinchera A., Braverman L. (1984 Jul). Environmental iodine intake and thyroid dysfunction during chronic amiodarone therapy. Ann. Intern. Med..

[5] Cohen-Lehman J., Dahl P., Danzi S., Klein I. (2010 Jan). Effects of amiodarone therapy on thyroid function. Nat. Rev. Endocrinol..

[6] Bogazzi F., Bartalena L., Martino E. (2010 Jun). Approach to the patient with amiodarone-induced thyrotoxicosis. J. Clin. Endocrinol. Metab..

[7] Bogazzi F., Bartalena L., Dell'Unto E., Tomisti L., Rossi G., Pepe P., Tanda M.L., Grasso L., Macchia E., Aghini-Lombardi F., Pinchera A., Martino E. (2007 Oct). Proportion of type 1 and type 2 amiodarone-induced thyrotoxicosis has changed over a 27-year period in Italy. Clin. Endocrinol..

[8] Kotwal A., Touchan B., Seetharaman K.Y., Haas R.A., Lithgow M., Malkani S. (2015). Mixed amiodarone-induced thyrotoxicosis refractory to medical therapy and plasmapheresis. J Endocrinol Metab.

[9] Conen D., Melly L., Kaufmann C., Bilz S., Ammann P., Schaer B., Sticherling C., Muller B., Osswald S. (2007 Jun 19). Amiodarone-induced thyrotoxicosis: clinical course and predictors of outcome. J. Am. Coll. Cardiol..

[10] Bourcier S., Coutrot M., Kimmoun A., Sonneville R., de Montmollin E., Persichini R., Schnell D., Charpentier J., Aubron C., Morawiec E., Bigé N., Nseir S., Terzi N., Razazi K., Azoulay E., Ferré A., Tandjaoui-Lambiotte Y., Ellrodt O., Hraiech S., Delmas C., Barbier F., Lautrette A., Aissaoui N., Repessé X., Pichereau C., Zerbib Y., Lascarrou J.B., Carreira S., Reuter D., Frérou A., Peigne V., Fillatre P., Megarbane B., Voiriot G., Combes A., Schmidt M. (2020 Jan). Thyroid storm in the ICU: a retrospective multicenter study. Crit. Care Med..

[11] Bartalena L., Bogazzi F., Chiovato L., Hubalewska-Dydejczyk A., Links T.P., Vanderpump M. (2018 Mar). 2018 European thyroid association (ETA) guidelines for the management of amiodarone-associated thyroid dysfunction. Eur. Thyroid J..

[12] Kotwal A., Clark J., Lyden M., McKenzie T., Thompson G., Stan M.N. (2018 Sep 6). Thyroidectomy for amiodarone-induced thyrotoxicosis: mayo clinic experience. J Endocr Soc.

[13] Simsir I.Y., Ozdemir M., Duman S., Erdogan M., Donmez A., Ozgen A.G. (2018 Oct). Therapeutic plasmapheresis in thyrotoxic patients. Endocrine.

[14] Upadhyaya V.D., Douedi S., Akula M., Chalasani K.K., Saybolt M.D., Hossain M. (2020 Mar). Amiodarone-induced thyroid storm causing sustained monomorphic ventricular tachycardia treated with plasmapheresis: a challenging clinical case. J. Med. Cases.

[15] Tonnelier A., de Filette J., De Becker A., Deweer S., Velkeniers B. (2017 Apr). Successful pretreatment using plasma exchange before thyroidectomy in a patient with amiodarone-induced thyrotoxicosis. Eur. Thyroid J..

[16] Kirkizlar H.O., Celik M. (2022 Aug 13). Therapeutic plasma exchange in hyperthyroidism prior to surgery. J. Endocrinol. Invest..

[17] Bouça B., Martins A.C., Bogalho P., Sousa L., Bilhim T., Gomes F.V., Coimbra É., Agapito A., Silva-Nunes J. (2022 Jan 1). Thyroid arterial embolization in a patient with congenital heart disease and refractory amiodarone-induced thyrotoxicosis. Eur. Thyroid J..

[18] Ozcan E.E., Dogdus M., Yilancioglu R.Y., Adiyaman S.C., Turan O.E. (2022 Mar 18). Invasive heart rate control as a salvage therapy in amiodarone-induced thyroid storm. Medeni Med J.

[19] Samaras K., Marel G.M. (1996 Sep). Failure of plasmapheresis, corticosteroids and thionamides to ameliorate a case of protracted amiodarone-induced thyroiditis. Clin. Endocrinol..

[20] Cappellani D., Papini P., Pingitore A., Tomisti L., Mantuano M., Di Certo A.M., Manetti L., Marconcini G., Scattina I., Urbani C., Morganti R., Marcocci C., Materazzi G., Iervasi G., Martino E., Bartalena L., Bogazzi F. (2020 Jan 1). Comparison between total thyroidectomy and medical therapy for amiodarone-induced thyrotoxicosis. J. Clin. Endocrinol. Metab..

[21] Rubio G.A., Koru-Sengul T., Vaghaiwalla T.M., Parikh P.P., Farra J.C., Lew J.I. (2017 Jun). Postoperative outcomes in Graves' disease patients: results from the nationwide inpatient sample database. Thyroid.

[22] Frank E.D., Park J.S., Watson W., Chong E., Yang S., Simental A.A. (2020 Aug). Total thyroidectomy: safe and curative treatment option for hyperthyroidism. Head Neck.

[23] Elnaggar M.N., Jbeili K., Nik-Hussin N., Kozhippally M., Pappachan J.M. (2018). Amiodarone-induced thyroid dysfunction: a clinical update. Exp. Clin. Endocrinol. Diabetes.

[24] Maqdasy S., Batisse-Lignier M., Auclair C., Desbiez F., Citron B., Thieblot P., Roche B., Lusson J.R., Tauveron I. (2016 Apr 1). Amiodarone-induced thyrotoxicosis recurrence after amiodarone reintroduction. Am. J. Cardiol..

[25] Czarnywojtek A., Warmuz-Stangierska I., Woliński K., Płazińska M., Kobylecka M., Kunikowska J., Stangierski A., Miechowicz I., Waligórska-Stachura J., Rewers A., Królicki L., Ruchała M. (2014). Radioiodine therapy in patients with type II amiodarone-induced thyrotoxicosis. Pol. Arch. Med. Wewn..

[26] Bogazzi F., Tomisti L., Ceccarelli C., Martino E. (2010 Jan). Recombinant human TSH as an adjuvant to radioiodine for the treatment of type 1 amiodarone-induced thyrotoxicosis: a cautionary note. Clin. Endocrinol..

[27] Yamato M., Wada K., Hayashi T., Fujimoto M., Hosomi K., Oita A., Takada M. (2018 Jan). Asociation between serum amiodarone and N-desethylamiodarone concentrations and development of thyroid dysfunction. Clin. Drug Invest..

