# Supplementary material for: Life-threatening amiodarone-induced thyrotoxicosis – Personalized approach to radical treatment
Source: Heliyon. 2024 Jul 18;10(14):e34850. doi: 10.1016/j.heliyon.2024.e34850 (PMC11327811; doi:10.1016/j.heliyon.2024.e34850)
Supplement: Multimedia component 1 [file mmc1.docx]

Supp. Table 1. Clinical data and results of thyroid function assessment, concertation of anti-thyroid antibodies at diagnosis of AIT, liver enzymes serum in 29 patients who respond well to medical treatment and were subsequently treated with 131-I to radically cure hyperthyroidism or prevent its recurrence after re-induction of amiodarone.

| Variable | Median (range)/no (%) | Normal range |
| --- | --- | --- |
| Gender, male | 38 (69.1) | - |
| Age, years | 64 (31-93) | - |
| TSH at diagnosis [uIU/ml] | 0.001 (0.001-0.12) | 0.27 – 4.20 |
| Free T4 at diagnosis [pmol/l] | 45.8 (8.9 – 120.0) | 12.0 – 22.0 |
| Free T3 at diagnosis [pmol/l] | 8.8 ( 3.8 – 49.3) | 3.1 – 6.8 |
| TRAb, [U/L] | 0.6 (0.1 – 11.8) | 0.0 – 1.0 |
| aTPO, [IU/ml] | 11.6 (0.01 – 199.0) | 0.0 – 34.0 |
| aTg, [IU/ml] | 14.1 (0.01 - 441.0) | 0.0 – 115.0 |
| Time of amiodarone use [months] | 22.8 (0.01 – 114.5) | - |
| Duration of hospitalization [months] | 0.42 (0.1-3.0) | - |
| Total time of ATD use [months] | 5.7 (1.0 – 20.0) | - |
| Total time of glucorticosteroids use [months] | 6.0 (0 – 15.5) | - |
| Use of rhTSH, no of patients | 1 (3) | - |
| Time between amiodarone cessation and 131-I therapy [months] | 7.0 (1 -27) | - |
| Time between 131-I therapy to conformation of eu- or hypothyroidism [months ] | 3.7 (1.0 – 14.0) | - |
